# Three-Dimensional Bioprinting of Astrocytes and Endothelial Cells to Direct Retinal Axon Growth and Vascularization

**DOI:** 10.1089/ten.tea.2024.0326

**Published:** 2025-11-14

**Authors:** Fatima E. Abukunna, Afnan M. Aladdad, Kiran J. McLoughlin, Khyathi Thallapureddy, Michael Vierra, Zoya Siddiqui, Karl E. Kador

**Affiliations:** Department of Ophthalmology and Department of Biomedical Sciences, University of Missouri-Kansas City, Kansas City, Missouri, USA.

**Keywords:** retina, bioprinting, retinal ganglion cell, vascularization, astrocyte, axon guidance, 3D printing

## Abstract

Retinal organoids (ROs) are currently used to study retinal development and diseases but cannot model glaucoma because they fail to form a nerve fiber layer (NFL) and optic nerve (ON). Utilizing three-dimensional bioprinting, ON head astrocytes (ONHAs) and vascular endothelial cells, both of which contribute to NFL development *in vivo* but are absent in ROs, were positioned at the center of scaffolds seeded with retinal ganglion cells (RGCs). In experiments using ONHAs isolated from developing retinas, polarization of RGC neurite growth increased by 43% while ONHA from adult retinas or astrocytes from the developing peripheral retina or developing cortex did not increase polarization above controls. Furthermore, RGC-seeded scaffolds increased both the number and rate of ONHAs migrating out from the printed center compared to scaffolds lacking RGCs, mimicking the migration pattern observed during retinal development. Finally, in scaffolds containing both ONHAs and endothelial cells, the endothelial cells preferentially migrate on and only form vascular tube structures on scaffolds also containing RGCs. These results suggest that recreating the developmental organization of the retina can recapitulate the mechanism of NFL development and retinal vascularization *in vitro.* This step is not only necessary for the development of retinal models of glaucoma but has the potential for translation to other parts of the central nervous system.

**Impact Statement:**

Using three-dimensional bioprinting to position the cells on an electrospun scaffold, we demonstrate that optic nerve (ON) head astrocytes from the developing retina are able to polarize retinal ganglion cell (RGC) growth to mimic the guidance found during development while astrocytes from the adult ON or from other locations are not able to direct this growth. We then show that these astrocytes will migrate out preferentially along the RGC axons, directing vascular endothelial cell growth and vasculature formation on our scaffold, recapitulating the mechanism of vascularization found in the retina.

## Introduction

Self-organizing retinal organoids (RO) which are able to form all retinal neuron cell types in the correct cell lamina have become a valuable model of retinal diseases as well as a source of cells for transplantation therapies.^1–5^ However, while human RO and some engineered retinal constructs are able to form the correct cell lamina, they are not able to be used as a model for glaucoma, the second leading cause of blindness, because they fail to properly form a retinal nerve fiber layer (NFL) and an optic nerve (ON).^6,7^

The guidance of retinal ganglion cell (RGC) axons to the ON in the retinal NFL is a function of extracellular matrix (ECM) based signals, cell-secreted neurotrophic factors and intrinsic signaling that regulate both axon outgrowth and directionality, with many of these signals being developmentally regulated.^8^ *In vitro*, isolated RGCs have been shown to respond to injections of guidance factors that are known to be involved in NFL guidance such as Netrin-1, Slit-2, and brain-derived neurotrophic factor (BDNF), and respond to ECM cues when cultured on substrates containing them.^9–11^ Despite the fact that it has been shown that RO forms a complex ECM when they are grown and that RGCs axon growth within the RO responds to these neurotrophic factors when added to the culture media through enhanced outgrow, these RO fail to form an NFL.^6,12^ These observations led us to hypothesize that failure to form an NFL may be caused by cells present during retinal development but which are absent during the RO formation. ROs are formed by the differentiation of embryonic or induced pluripotent stem cells (iPSCs) that are directed to a retinal progenitor cell state and are capable of differentiating into any retinal neuron.^13,14^ However, it has previously been shown that ON glial precursor cells, which originate in the brain and vascular cells are both involved in the secretion of signals found in NFL guidance.^15,16^ Interestingly, many of the cells involved in the formation of the retinal vasculature are also directly related to the cell-secreted factors that are involved in the guidance of the RGC axons. These axons form fasciculated neuron bundles that are guided into the ON and then provide the track for the migration of ON head astrocytes (ONAs) which in turn secrete soluble factors to guide the migration of vascular endothelial cells from the ON to vascularize the inner neural retina.^17^

In tissue engineering, physical as well as soluble or immobilized biochemical cues have been used to direct neuron growth in engineered constructs.^18–22^ We have previously developed a radial electrospun scaffold capable of directing RGC growth to mimic that of the retinal NFL; however, while RGC axons *in vivo* are polarized toward the ON head, the RGCs cultured on these scaffolds failed to have their axons polarized toward the scaffold center.^11,23^ Immobilization of Netrin-1 onto these scaffolds enhances this polarization, but the overall number of RGCs remained less than 55% polarized toward the scaffold center. In other studies, HEK cells modified to secrete Netrin-1 have been positioned in a collagen matrix to direct neuron growth, while more recently newly developed three-dimensional (3D) bioprinting techniques have been used to position cells in separate layers in order to direct cell growth between the two cell layers where synapses can be formed.^24^

Similar techniques, such as the use of electrospun fibrous scaffolds and immobilized or secreted factors such as vascular endothelial growth factor (VEGF), fibroblast growth factor (FGF), and platelet-derived growth factor (PDGF) have been used to direct vascular growth in tissue-engineered constructs.^25–28^ 3D printing techniques have also been utilized to create vasculature by printing perfusable tubes into the tissue-engineered constructs for delivery of fresh media during culture before being seeded on the luminal side of the tube with vascular endothelial cells.^29^ This method allows for the growth of constructs with higher cell densities because the thickness of the tissue-engineered constructs is limited by nutrient diffusion. These 3D-printed vascular structures are limited in both their complexity and size by the resolution of the 3D printer which in most studies is several hundred microns in diameter. The development of digital light processing 3D printers which utilize specific wavelengths of light to crosslink scaffold materials in place has increased the resolution of the printed structures; nevertheless, the printed vasculature still is not able to match vessel size found in the native tissue.

In this study, we will demonstrate that 3D printing ONH glial cells in combination with electrospun scaffolds in order to recreate the organization of the developing retina can be used to direct and polarize RGC growth *in vitro.* We will demonstrate that this guidance is dependent on the location and developmental time point from which the glia are isolated and demonstrate that the cells responsible for this guidance are the ONH astrocytes. We will then recapitulate the mechanism combining RGCs, ONHA, and vascular endothelial cells which link axon guidance and vascularization in the inner retina. We will demonstrate that the 3D-printed ONHA will preferentially migrate along RGCs while also demonstrating that glial cell migration can be used to direct endothelial cell migration and vascularization within the constructs. This model not only represents an important step in creating retinal constructs for the study of glaucoma but also demonstrates a potential method for vascularizing CNS tissue-engineered constructs.

## Materials and Methods

### RGCs isolation

RGCs were isolated using a two-step immunopanning method described previously.^23^ Retinas were dissected from postnatal (postnatal days 2–5) Sprague Dawley (SD) rats and digested in papain (165 U/L). Mechanical trituration was used to dissociate retinas into a single-cell suspension which was then depleted of macrophage and endothelial cells using an antirat macrophage antibody (Accurate Chemicals), and RGCs were isolated using an anti-Thy1 antibody (T11D7, American Type Culture Collection [ATCC]). RGCs were cultured on laminin-coated substrates in media composed of: neurobasal media, supplemented with insulin, sodium pyruvate, penicillin/streptomycin, glutamax, *N*-acetyl cysteine, triiodothyronine, transferrin, progesterone, sodium selenite, putrescine, forskolin, B27, BDNF, and ciliary neurotrophic factor (CNTF). Concentrations for each component can be found in the Supplementary Data.

### Astrocytes isolation

Astrocytes were dissected from the ON of postnatal day 2 (P2) and adult (8 weeks) SD rats or from the retina, excluding the ON, of P2 SD rats. For ONH astrocytes, tissues were minced and digested with 165 units of papain for 30 min and placed in culture cultured in a poly-d-lysine (PDL) coated T75 flasks (2 ONHs per flask) with Dulbecco’s Modified Eagle’s Medium (DMEM) high glucose, 10% fetal bovine serum (FBS), 1% penicillin/streptomycin, and heparin-binding epidermal growth factor (HBEGF) 10 ng/mL with medium change every other day. The ONH astrocytes formed a monolayer within a week of culture and passaged every 3–5 days. Astrocytes from the surrounding retina were isolated following an RGC isolation experiment by taking the unbound retinal cells depleted of macrophage and endothelial cells and placing them in culture in a PDL-coated T75 flask with the media described above. Because retinal neurons are postmitotic, the expanded cells were collected as retinal astrocytes, but no further purification for these or either of the primary ONH astrocyte populations was made.

### Astrocyte cell line culture

Type 1 (clone C8-D1A) and Type 2 (clone C8-S) astrocytes,^30^ were purchased from ATCC and cultured on PDL-coated T75 flasks with DMEM high glucose media supplemented with 10% FBS and 1% penicillin/streptomycin. Cells were passaged every 3–5 days.

### Purification of different cell subtypes from primary ONHA

Subpopulations from the P2 ONHA astrocytes were purified using established immunopanning protocols.^31,32^ Briefly, passage 2 P2 ONHA was trypsinized, centrifuged, and resuspended in panning buffer (0.02% BSA and 5 µg/mL insulin in dPBS containing calcium, magnesium, and glucose) and split into two equal volumes. Cells were then purified following one of two sequences with unbound cells moving to each subsequent plate and bound cells trypsinized and collected. Sequence 1, cells were incubated for 10 min on a plate coated with Griffonia Simplicifolia Lectin, 10 min on a goat anti-rat antibody coated plate, 20 min on a plate coated with anti-CD45 antibody, 15 min on a plate coated with anti-O4 antibody and 40 min on a plate coated with anti-integrin β5 with cells which did not bind to any plate also collected and labeled flow through. Sequence 2, cells were incubated for 30 min on a plate coated with anti-C5 antibody and for 30 min on a plate coated with anti-CD13. Flow-through cells from the second sequence were discarded. Cells isolated from each of these plates were expanded in DMEM high glucose media containing 10% FBS and 1% penicillin/streptomycin. Catalog numbers for the antibodies used for purification are listed in the Supplementary Data.

### Purification and culture of human endothelial colony-forming cells

Primary human cord blood mononuclear cells (Stem cell technology# 70007.1) seeded at a density of 35–50 × 10^6^ cells/well in a collagen-1 precoated six wells plate. The cells were cultured with Endothelial Cell Growth Medium-2 (EGM-2) media (Lonza) for 14 days with daily medium change for the first 7 days and every other day afterward. Endothelial colony-forming cells (ECFC) colonies appeared on day five and were visible at 10× magnification. Cell sorting was conducted using a Wolf low-pressure cell sorter was used to purify cells for CD31 positive and CD45 and CD90 negative immunoreactivity to exclude hematopoietic and mesenchymal. The negative selection was conducted via immunopanning followed by positive selection via fluorescence activated cell sorting (FACS). Purified ECFCs were subcultured every 2–4 days. Catalog numbers for the antibodies used for purification are listed in the Supplementary Data.

### Electrospinning

Radial scaffolds were electrospun, as mentioned previously.^23^ Briefly, polylactic acid (Purasorb PDL10, Corbion) was dissolved in hexafluoroisopropanol at a concentration of 6.6% and the solution was then pumped at a rate of 2 mL/h through a 20-gauge stainless steel needle that was charged at 15 kV. The target was a radial collector made of a 1.8-cm aluminum foil-covered cup with an 18-gauge copper wire in the center grounded to the same ground as the aluminum foil, placed 12 cm from the needle. Scaffolds were sterilized for 20 min using a germicidal UV light.

### Bioink optimization

RGCs or P2 ONHA were suspended within: (i) growth factor reduced matrigel; (ii) 4% collagen-1; (iii) 1% sodium alginate in dPBS with Ca^2+^ or Mg^2+^ but containing insulin; (iv) 1% sodium alginate modified to contain arginyl-glycyl-aspartic acid (RGD) peptides in dPBS with Ca^2+^ or Mg^2+^ but containing insulin and 50/50 mixtures of each of these inks. Inks were plated into the glass bottom 24 well culture plates and cultured for 3 days. Following culture, samples were analyzed using live/dead staining and analyzed for survival, cell spreading, and RGCs neurite extension.

### Media optimization

RGCs were seeded into laminin-coated multiwell plates and cultured for 10 days with: (i) RGC media; (ii) RGC media including HBEGF and N2 supplement; (iii) RGC media including HBEGF, N2, and EGM-2 kit growth factors; or (iv) EGM-2 media including growth factors and HBEGF, N2, BDNF and CNTF (the growth factors found in the optimized RGC media). After 10 days, RGCs were analyzed using live dead staining.

### Bioprinting

Astrocytes with or without ECFCs were cultured as stated above and harvested using TrypLE™ Express Enzyme. Cells were resuspended in matrigel at 4°C at a concentration of 1–1.5 × 10^6^ cells/mL and mixed thoroughly to ensure cell homogeneity. The matrigel matrix cell solution was loaded into a 5 mL syringe, and a 25 G flat tip needle was attached without introducing air bubbles and placed in the precooled printhead. Bioprinting was conducted using an extrusion-based Allevi 3 bioprinter (3D systems). Printheads, syringes, nozzles, and matrigel were precooled to 4°C. Printing parameters: Structure: 3 mm diameter cylinder with 0.5 mm height, blunt tip needle size 25 G, printhead temperature 4°C, printing speed 6 mm/s print bed 37°C, pressure 7.5–10 psi, post-printing incubation 10–15 min at 37°C. After post-printing incubation, pre-warmed media was added and the sample was placed in culture.

### Neurite guidance experiments

Astrocytes were printed at the center radial scaffolds and cultured overnight in RGC media with 2 µg/mL laminin-1 (Cultrex). RGCs were isolated and seeded at a density of 5,000 RGCs/scaffold on the scaffold surrounding the printed astrocytes. Samples were cultured for 48 h in fresh RGC media before being fixed and stained for β3-Tubulin. Cells were counted for growth toward the scaffold center, away from the scaffold center, or in both directions for all neurites greater than two cell bodies in length. Because of mechanical damage that occurred to some scaffolds following the 3D printing of the astrocytes resulting in failures of the scaffolds and an uneven group number, including some groups with only a single scaffold surviving within an experimental set, all samples across experiments were grouped into a single set so that each sample was weighted equally for statistical analysis.

### Migration assays

Overall, 1 cm thick polydimethlsiloxane (PDMS) discs were punched out using a 12 mm biopsy punch and cut in half. Half of each PDMS disc was placed over a laminin-coated scaffold and 20,000 RGCs were seeded on the uncovered half of the scaffold. The PDMS disc was removed after 24 h and the RGCs cultured for a further 24 h. Forty-eight hours after RGC seeding, astrocytes or both astrocytes and ECFCs were printed at the center of the scaffold. Samples were cultured for a further 10 days before being fixed and stained for β3-Tubulin, S100β, CD31, ZO-1, and/or 4′,6-diamidino-2-phenylindole (dapi). Astrocyte migration assays were measured using samples cultured in RGC media supplemented with HBEGF and N2. ECFC migration assays were cultured first in RGC media supplemented with HBEGF and N2 before media optimization. Because of mechanical damage that occurred to some scaffolds following the 3D printing of the astrocytes resulting in failures of the scaffolds and an uneven group number, including some groups with only a single scaffold surviving within an experimental set, all samples across experiments were grouped into a single set so that each sample was weighted equally for statistical analysis.

### Live and dead staining

Following culture, cells were stained with calcein-AM and propidium iodide, both at a concentration of 1:500 in the media used for the experiment, and incubated for 30 min. Following incubation, cells were imaged using a Leica DMI6000 inverted fluorescence microscope with live cells being labeled with green fluorescence and dead cells labeled red. ImageJ software was used to count the cells. The percentage of viable cells was then calculated as (number of live cells/[total number of live + dead cells in the image]) × 100. The results were obtained from three different experiments with 3–4 technical replicates. Nine images per replicate were counted.

### Immunocytochemistry

Samples were fixed with 4% paraformaldehyde for 30 min, blocked, and permeabilized with blocking buffer (10% FBS, 0.5% Triton X-100 in 1X PBS) for 2–4 h. The primary antibodies S100β (1:500, Abcam-b52642 and 1:300, Invitrogen-710363), β3-Tubulin (1:500, Synaptic Systems-302 306) and CD31 (1:500, Novus Biologicals-NBP2-80641) and secondary antibodies goat antirabbit 488-ab150077, goat antimouse 568-ab175473 and were diluted to 1:500 using 2% FBS and 0.3% Triton X-100 in 1× PBS. Samples were incubated overnight at 4C°, and DAPI at 1:1000 was added for 5 min. Leica CTR6500 fluorescence, SP5, and Stellaris 8 confocal microscopes were used for imaging.

### Vascular analysis

Entire scaffolds were imaged and the CD31 labeled images were divided into quarters for analysis. Images were analyzed for vascular formation using the angiogenesis analyzer plugin for ImageJ. This plugin automatically identifies and analyzes multiple angiogenesis-related parameters including branching length, number of junctions, number of branches, number of meshes and extremities, and nodes.^33,34^ The vascularized area was calculated by dividing the vascularized mesh area by the total area examined. Data presented as mean ± standard deviation of 22 images from at least three independent experiments.

## Results

### Bioink optimization

Bioinks are solutions containing one or more biomaterials that act as the mobile phase and cell carrier during the 3D printing process and in many cases become the material in which the cells are grown.^35^ Because bioinks are able to include biochemical signals that can direct the growth and differentiation as well as the health and survival of the cells being printed, optimizing bioink characteristics and parameters is essential in fabricating 3D tissue constructs.^36^ While RGCs will not be printed in these constructs, because their axons will grow into the printed structure it is necessary to analyze their interaction with each bioink in addition to that of the astrocytes. RGCs isolated from early postnatal SD rats and astrocytes isolated from the P2 ON head of SD rats were printed using a 22 g needle in four previously published bioinks: undiluted matrigel, 4 mg/mL collagen-1, 1% alginate, and 1% alginate-modified with RGD cell binding domain peptides. In addition, RGCs and ONHAs were printed and cultured in mixtures of these same bioinks. In this study, printing parameters were based on published parameters from the manufacturer.^37^ Because others have shown that cell viability for 3D-printed astrocytes does not change based on the ECM components in the bioink when the printing parameters are identical, these studies focused on cell morphology to determine the interaction for both cell types.^38^ Following 3 days in culture, RGCs and astrocytes were stained and imaged using the viability dye calcein-AM (Fig. 1). Living RGCs and astrocytes were observed in all samples, however, RGCs did not extend neurites and astrocytes remained rounded and did not stretch in samples which did not contain either matrigel or collagen-1 with RGCs printed in matrigel containing the greatest neurite extension. Due to the increased compatibility of both RGCs and ONHAs, matrigel was used as the bioink in all subsequent experiments.

**FIG. 1.**
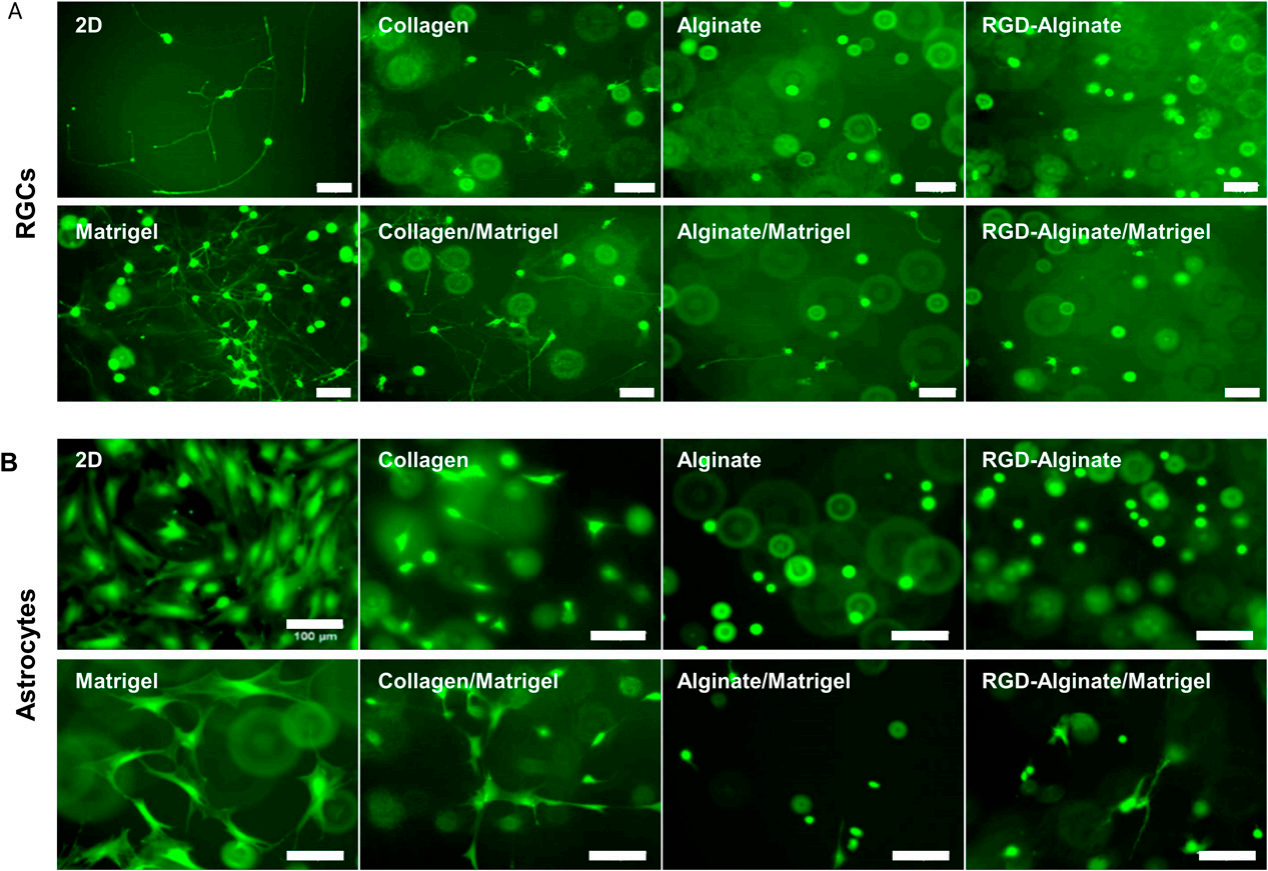
Cell-Bioink biocompatibility. RGCs **(A)** and astrocytes **(B)** were cultured for 3–4 days in different bioinks, including 4 mg/mL collagen 1, 1% alginate, 1% alginate modified with RGD peptides, Matrigel, and mixtures of these four components, and compared to the growth on 2D cultures. When stained with the cell viability dye calcein-AM, RGCs were observed to extend neurites, and astrocytes were observed to spread on bioinks containing matrigel or collagen with the greatest neurite outgrowth occurring on RGCs seeded on matrigel. Cells seeded on Alginate or Alginate modified with RGD peptides were observed to be rounded and did not interact with the bioink material. All experiments were conducted in RGC-optimized media. Scale bar = 100 µm. 2D, two-dimensional; RGCs, retinal ganglion cells.

### RGC axon guidance

During development, RGC axons in the retinal NFL (RNFL) are directed to the ON head and then to the brain by a range of soluble and matrix-associated signals.^8^ 3D printing techniques have also been utilized to create vasculature by printing perfusable tubes into the tissue-engineered constructs for delivery of fresh media during culture before being seeded on the luminal side of the tube with vascular endothelial cells However, RGCs do not polarize their growth when cultured *in vitro* as a 2D cell culture or as part of an RO. While ROs follow the developmental stages of the retina which includes the differentiation of all retinal neurons within the correct cell laminas, they do not contain the cells of the ONH that migrate into the retina via the ON. In order to introduce the missing secreted factors from these cells, astrocytes were printed at the center of radial electrospun scaffolds capable of directing RGC axon growth along the radial fiber but not polarizing that growth toward the center. To determine whether these secreted factors were common to all astrocytes or only to a specific subset of astrocytes, five populations of astrocytes were tested. These included primary ONHA isolated from the P2 developing retina of an SD rat, primary retinal astrocytes isolated from the retina not including the ON from the P2 SD rat, primary ONHA isolated from an adult (16 week) SD rat, and two mouse cortical astrocyte cell lines derived from a postnatal mouse—one composed of type 1 astrocytes and the other composed of type 2 astrocytes. All groups were compared to a matrigel alone control (Fig. 2A–C). After an initial 24 h culture, RGCs were seeded surrounding the printed astrocytes and cultured for a further 48 h with all cultures being conducted in RGC-optimized media supplemented with HBEGF and N2 supplement. The RGCs were then stained for β3 tubulin, and the polarity of their neurites measured as directed toward the center, directed away from the center, or extending in both directions for all neurites greater than two cell bodies in length (Fig. 2D). Two cell bodies in length was chosen because all neurites of this length or greater have been shown to stain positive for axonal marker SMI-312.^39^ While no significant differences between controls containing matrigel alone and matrigel printed with ONHA from the adult animal, retinal astrocytes from the developing retina or cortical astrocytes were observed, a significant increase in the number of RGCs with neurites polarized toward the scaffold center and a significant decrease in the number of RGCs extending neurites away from the center were observed when the scaffolds contained printed with ONHAs from the developing P2 retina (Fig. 2E).

**FIG. 2.**
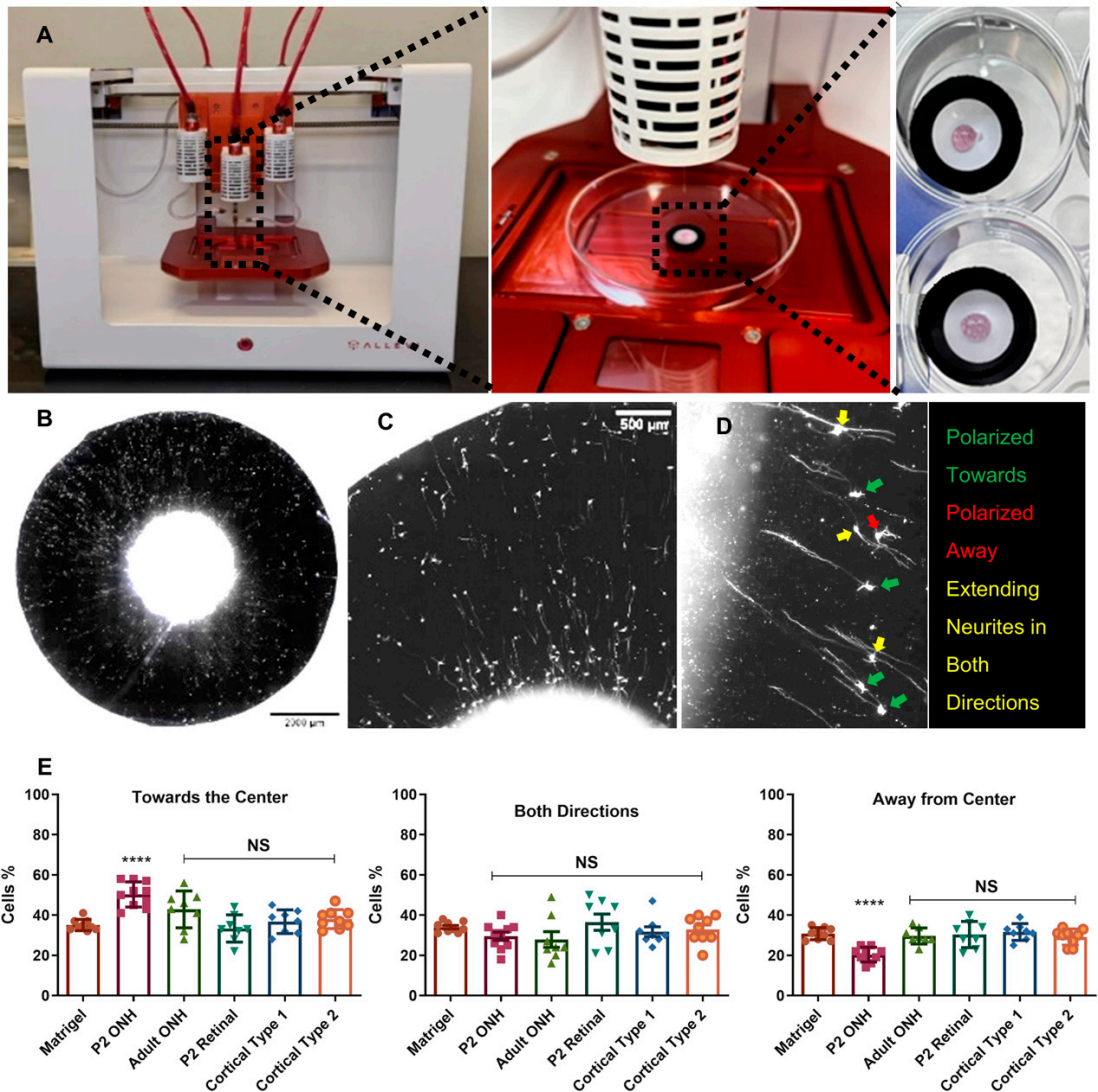
Astrocytes printed at scaffold center increase polarization of RGCs axon growth. **(A)** 3D bioprinting of astrocytes in a matrigel bioink onto radial scaffold. Matrigel with astrocytes on the center of the scaffold. **(B** and **C)** Representative fluorescence images show RGCs cultured around the bioprinted astrocytes on scaffolds. **(D)** Representative image of counting of RGCs stained with β-III tubulin according to their direction toward the center, away from the center, or in both directions. While this image is taken next to the printed area, RGCs were quantified across the entire scaffold. **(E)** Quantification of the RGC growth based upon the astrocyte. Only astrocytes isolated from the P2 ONH showed a significant increase in polarization. All experiments were conducted in RGC-optimized media supplemented with HBEGF and N2. Scale bar = 2,000 µm **(C)** and 500 µm **(D)**. Data are shown as the mean ± standard deviation. One-way analysis of variance (ANOVA) with Bonferroni correction (****p < 0.0001). HBEGF, heparin-binding endothelial growth factor; P2, postnatal day 2; ONH, optic nerve head.

Because the primary P2 ONHA populations are a mixed population that includes other ONH cells, studies were conducted to identify the cell type ultimately responsible for the increase in RGC polarization. The P2 ONHA populations were expanded and then sorted using two established immunopanning protocols for the purification of central nervous system glia. Using this protocol, endothelial cells, macrophages, microglia, pericytes, astrocytes, and nonbinding flow-through cells were purified. The purification of oligodendrocytes was also attempted, however, none were identified within the cell population. Printing each of these cell types at the center of radial scaffolds demonstrated that only the astrocyte population increased RGC polarization (Fig. 3).

**FIG. 3.**
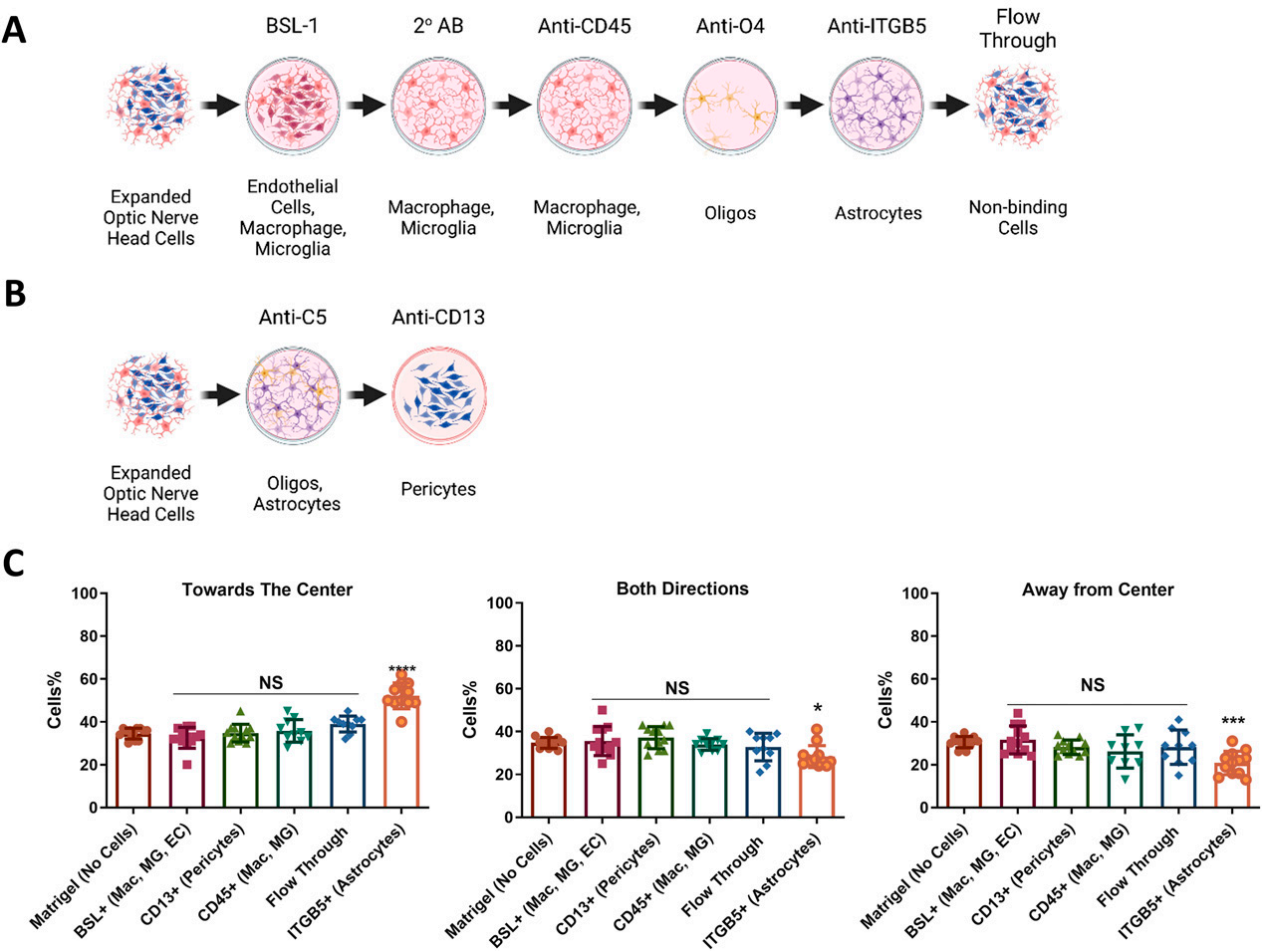
Purification of cell subtypes from P2 ONHA cells. P2 ONHA cells were purified by two established immunopanning protocols to isolate different cells from the optic nerve head. Cells from each plate, except the anti-O4 plate which had no cells bound, were expanded and then printed on scaffolds and used in RGC guidance assays. Only cells bound to the integrin β5 (ITGB5) plate demonstrated an increase in guidance (*p < 0.05, ***p < 0.001, ****p < 0.0001). All guidance experiments were conducted in RGC-optimized media supplemented with HBEGF and N2. ONHA, optic nerve head astrocytes. Created with Biorender.

### Migration of astrocytes toward the RGCs

Optic disc progenitors produce the astrocytes that are located at the junction of the optic cup and stalk, and these astrocytes are involved in vascularizing the developing retina.^40,41^ The astrocytes enter the developing retina from the ON head following the RGCs axon before retinal angiogenesis occurs.^17,40,42,43^ Aligned and radially aligned electrospun scaffolds, similar to those used by us to replicate the alignment of the NFL of the retina, have previously been shown to induce cell migration in the absence of other biochemical cues.^44–46^ To determine whether astrocytes preferentially migrate along RGC axons *in vitro*, one-half of each radial scaffold was covered with a polydimethylsiloxane (PDMS), and the other half was seeded with RGCs. A PDMS mask was used to restrict the placement of the RGCs rather than 3D printing of the RGCs as the PDMS mask can restrict cell placement on the cm scale where 3D printing places cells on the 100 µm scale making the PDMS mask more efficient this purpose and limiting the time the RGCs were outside of pH control. After 8 h of culture, the PDMS mask was removed and the RGCs were allowed to extend their neurites for an additional 48 h of culture. At that point, ONH astrocytes were printed onto the scaffold center in matrigel. The cells were then cultured in regular RGCs medium supplemented with HBEGF and N2 for 10 days. At the end of culture, all samples were stained for β3 tubulin and S100β (Fig. 4A, B). While astrocytes were observed to grow and migrate on both halves of the scaffolds, a significantly larger percentage of astrocytes were observed to migrate on the half seeded with RGCs (Fig. 4C). In addition to preferentially migrating on the RGC-seeded half of scaffolds, astrocytes were observed to migrate further than on the half of the scaffold with RGCs, migrating an average distance of 3156 µm (Fig. 4D) at a rate of 225 µm per day. Significance was established using Welch’s two-sided *t*-test due to the samples having unequal variances.

**FIG. 4.**
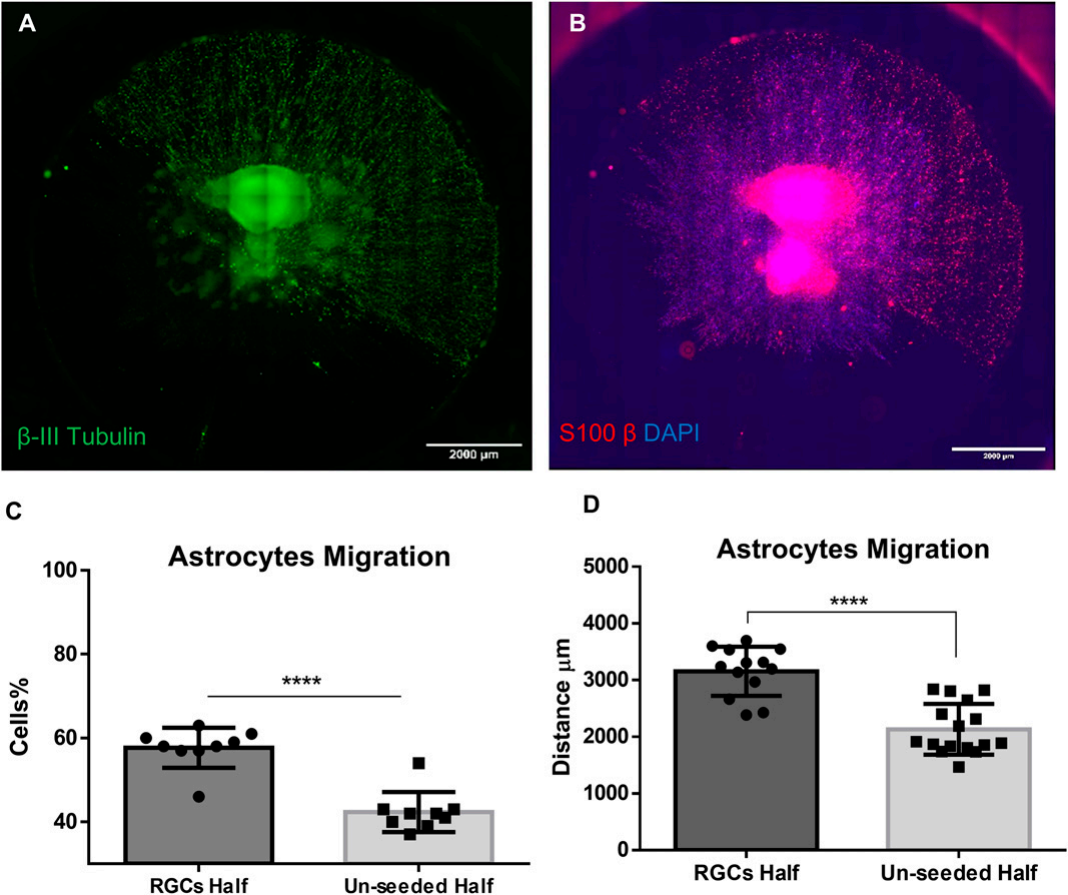
Astrocytes grow and migrate preferentially on RGCs. RGCs were seeded on half of the radial scaffold and grown for 48 h at which point P2 ONHA were printed in matrigel at the scaffold center. Cells were cultured together for 10 days in RGC-optimized media supplemented with HBEGF and N2. RGCs, labeled with β-III tubulin **(A)**, were observed to extend their neurites across half of the scaffold while astrocytes, labeled with S100β **(B)** were observed to preferentially migrate on the RGC half of the scaffold **(C)**. Astrocytes were also found to migrate further on the RGC-seeded half of the scaffold than the half coated with laminin alone **(D)**. Scale bar = 2000 µm in **A** and **B**. Data were analyzed using *t* tests and presented as mean ±  standard deviation. ****p < 0.0001.

### Selection of culture medium composition and RGCs survival

Proper selection of media components is essential for coculturing more than two cell types, especially when communication via soluble factors and extracellular vesicles are involved.^47^ The present study utilized three different cell types: RGCs, ONH astrocytes, and ECFCs, a vascular progenitor cell that has previously been used to generate retinal vasculature in models of retinal ischemia.^48,49^ Our initial experimental studies on RGC polarization, astrocyte migration, and ECFC migration assays were conducted using an optimized defined RGC growth media that has previously been used for the culture of both RGCs and astrocytes.^50^ This neurobasal-based media is supplemented with insulin, glutamax, sodium pyruvate, B27, *N*-acetal cysteine, triiodo-thyronine, Sato supplement, forskolin, BDNF, and CNTF with exact concentrations provided in the Supplementary Data. However, the viability, purity, and growth of ECFCs have previously been tested using different media with the optimal environment for ECFC growth being a defined medium containing vascular endothelial growth factor (VEGF), fibroblast growth factor 2 (FGF2), and epidermal growth factor (EGF).^51^ More recently the commercially available EGM-2 Bullet kit media which is a defined media containing growth factors that support the growth and angiogenesis of ECFCs, has been used for their culture.^52^ Because RGCs are postmitotic cells with the lowest viability of the three cell types to be used in the coculture, studies were conducted to examine RGC viability in four growth media: (i) Regular RGCs medium as a control media; (ii) RGCs medium, supplemented with N2 supplements and HBEGF to support astrocytes growth^31^; (iii) RGCs medium+ N2 supplements + HBEGF+ EGM-2 Bullet kit growth factors; and (iv) EGM-2 medium + N2+ HBEGF+ RGCs medium growth factors. After 10 days in culture, the RGCs were stained with calcein-AM and propidium iodide to identify living and dead cells respectively. ImageJ was used to determine the percentage of viable cells with significance determined using a one-way ANOVA with Bonferroni’s post hoc testing. Although RGCs survival was observed in all culture mediums examined (Fig. 5A), the highest viability was observed when EGM-2 Bullet kit growth factors were added to the RGC-optimized media (Fig. 5B). This media will be referred to as coculture optimized media in the following.

**FIG. 5.**
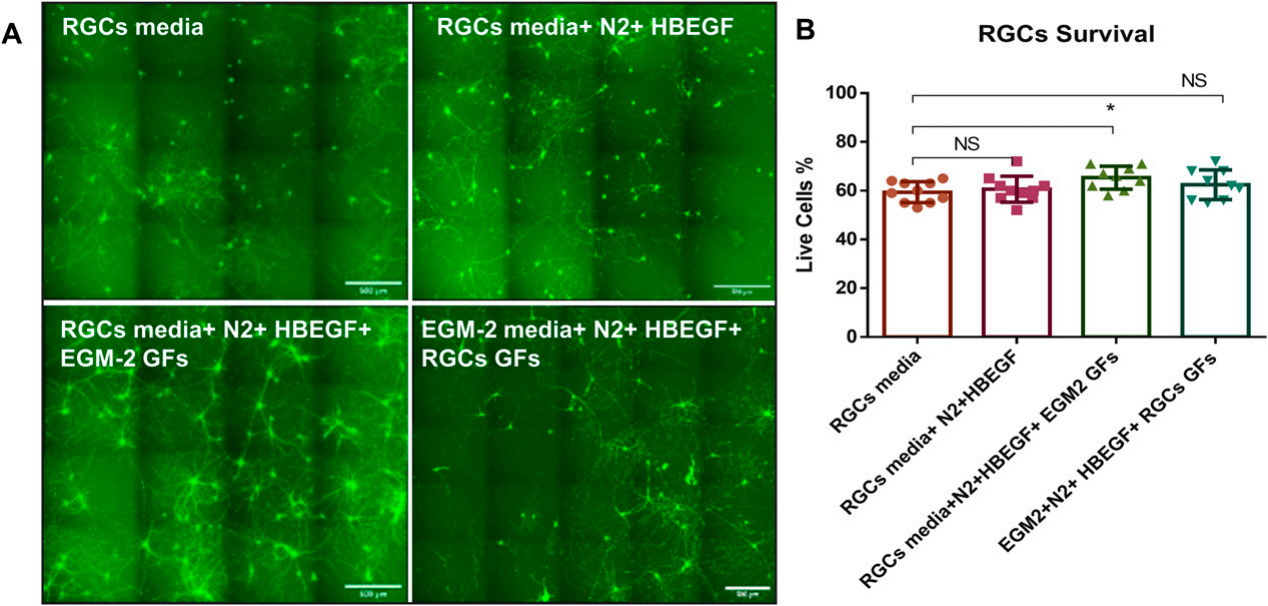
RGC survival in media selection. RGCs were seeded in four different media to analyze the effect of growth factors used to support endothelial cell growth on RGC viability in order to determine a media for their coculture. Viability was examined by live/dead staining following 10 days in culture **(A)**. While RGCs survival was similar in each media, fewer neurites were observed when the base media was switched from the neurobasal-based RGC growth media to the EGM2-based media optimized for vascular endothelial cell culture **(B)**. Based upon these results RGC media supplemented with N2, HBEGF, and EGM2 growth factors was used for cocultures with vascular endothelial cells. Significance was determined by one-way ANOVA with Bonferroni’s multiple comparisons test *p < 0.05. Scale bar = 100 µm.

### Endothelial cell migration

The developing retina is vascularized by angiogenesis in which endothelial cells sprout from preexisting blood vessels. In most tissues, endothelial cell migration is directed by macrophages,^53^ however in the retina, as in other parts of the central nervous system, this migration is guided by astrocytes.^17^ To determine if endothelial cells follow a similar mechanism *in vitro*, RGCs were again seeded on radial scaffolds using a PDMS mask to ensure seeding on half of the scaffold. After RGC seeding, ONH astrocytes, and ECFC were printed in growth factor reduced matrigel at the scaffold center. Following two weeks in culture in RGCs media supplemented with HBEGF and N2 supplement, an increased number of ECFCs migrating from the scaffold center onto the RGC-seeded side of the scaffold was documented by CD31 staining (Fig. 6A, B). Under these conditions the observed number of ECFCs migrating out of the printed matrigel at the scaffold center was small and the cells migrating were not forming continuous tubes (Fig. 6C, D). However, when RGCs/astrocytes/ECFCs were cocultured in coculture optimized media which included RGC growth media supplemented with N2, HBEGF, and the EGM-2 growth factors, ECFCs were observed to migrate across the entire scaffold surface though still showing a preference for the RGC half of the scaffold. More importantly, only ECFCs migrating on the RGC side of the scaffold were observed to form continuous tubes (Fig. 6E). Further examination of these vascular tubes indicated the presence of the tight junction protein ZO-1 along the cell membranes that colocalized with the CD31 expression (Fig. 6F).

**FIG. 6.**
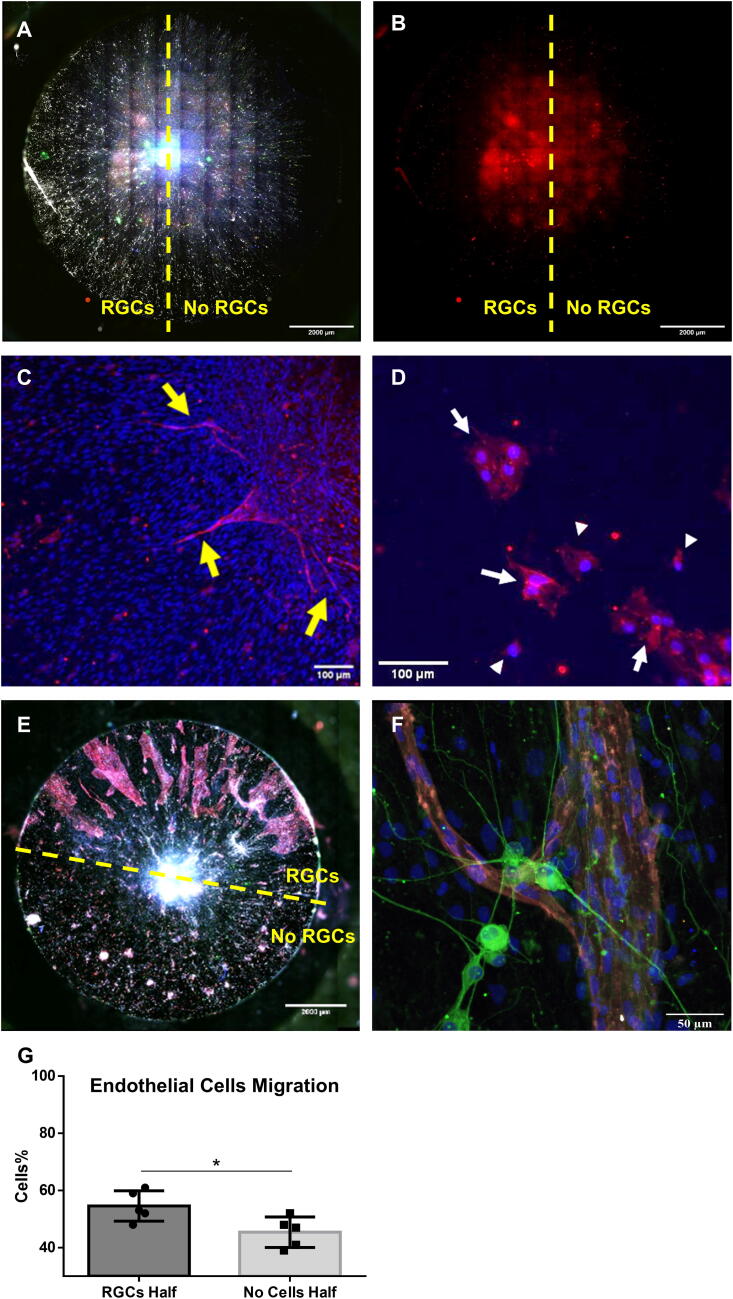
Endothelial cell migration. ECFCs printed with P2 ONHA at the center of each scaffold were observed to migrate preferentially on the RGC side of scaffolds **(A** and **B)**. While a leader follower migration pattern was observed as ECFCs exited the printed matrigel (C, *yellow arrows*) these cells migrated only as single cells (*white arrowheads*) or unorganized cell groups (*white arrows*) when cultured in RGC-optimized media supplemented with HBEGF and N2 **(D)**. When cultured in coculture optimized media (RGC-optimized media supplemented with HBEGF, N2, and ECM-2 growth factors), ECFCs were observed to create complex tube structures but only on the RGC-seeded half of the scaffold **(E)** including cells within the tube structures forming tight junctions **(F)**. Cell migration preference for ECFCs was calculated for scaffolds cultured in RGC media **(G)**. Scaffolds were stained with: β-III tubulin (RGCs) in *gray*, S100 β (astrocytes) in *gr*een, CD31 (ECFCs) in *red* and dapi to label all nuclei (A + E): for CD31 (*red* channel from A) **(B)**; for CD31 in red and dapi in *blue* (C + D); β-III tubulin in *green*, ZO-1 (tight junctions) in *gray*, CD31 (ECFCs) in *red* and dapi **(F)**. The *yellow* line marks the boundary of RGC seeding. **G** is presented as mean ±  standard deviation. Scale bar: **A**, **B**, and **E** = 2000 µm, **C** and **D** = 100 µm, **F** = 50 µm. *p < 0.05. ECFCs, endothelial colony-forming cells; ECM, extracellular matrix.

In order to determine the complexity of the vasculature formed in this coculture system, samples with RGCs seeded across the entire scaffold were utilized and the resulting vascularization formed across the entire scaffold (Fig. 7A, B) was imaged using confocal microscopy. The CD31 signal images were skeletonized using ImageJ software. (Fig. 7C) before being analyzed using the Angiogenesis Analyzer plugin. Vascular networks were quantified for the number of junctions, extremities, nodes, branches, and the percentage of vascularized area within fields of view of vascularized portions of scaffolds (*n* = 20). No measurements could be obtained for the controls for comparison because of a lack of vascularization in samples cultured in RGC growth media without EGM-2 growth factors or on scaffolds cultured without RGCs (Table 1).

**FIG. 7.**
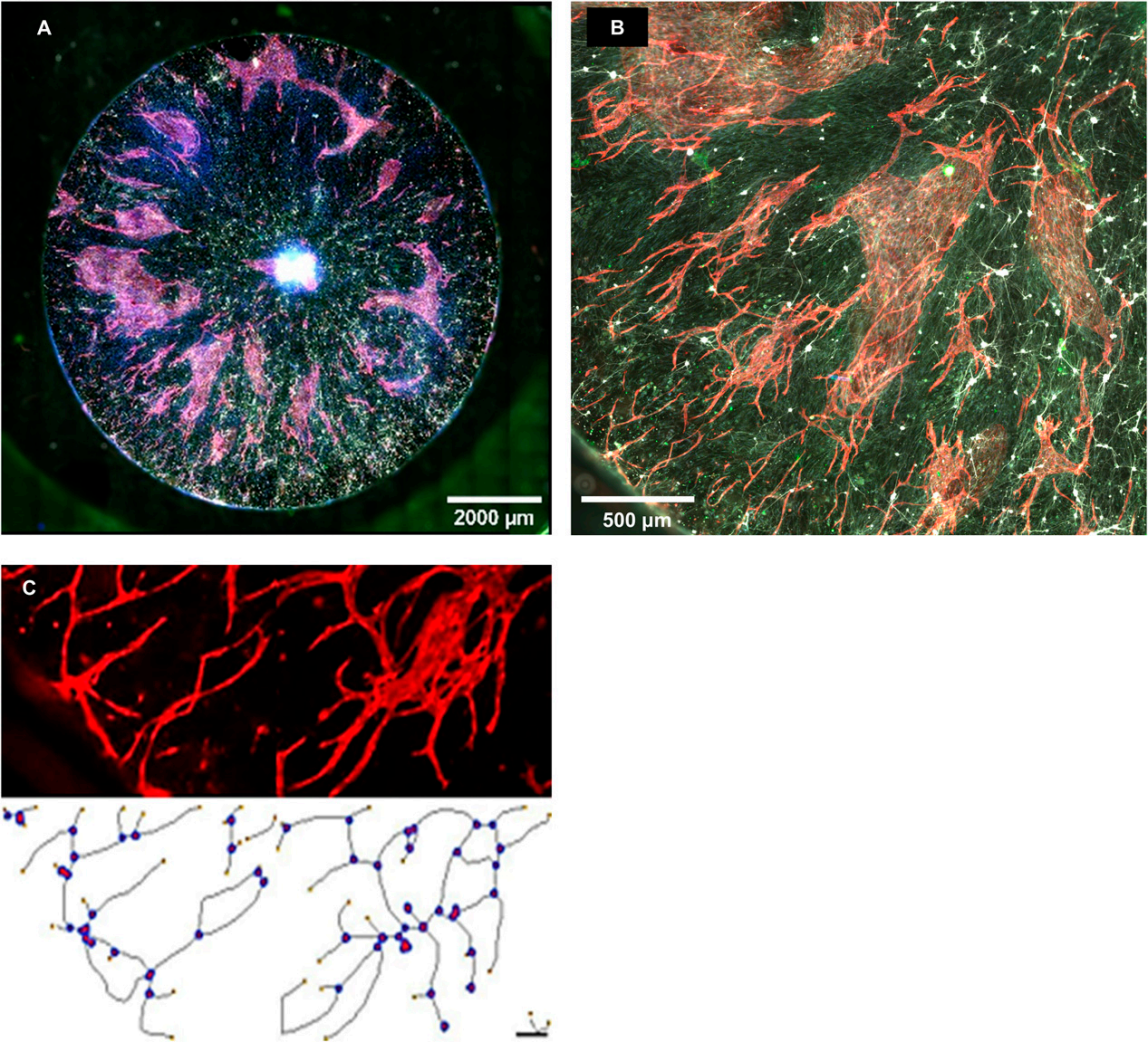
Vascularization of the engineered retinal-like construct. Scaffolds seeded with RGCs on the entire scaffold showed large areas of vascularization across the entire scaffold when cultured in the coculture optimized media containing HBEGF, N2, and EGM-2 growth factors **(A** and **B)**. CD31 stained images were skeletonized and angiogenic parameters were quantified with the ImageJ Angiogenesis Analyzer plugin. In this image, *red points* surrounded by a *blue circle* indicate nodes; the *black lines* surrounding nodes are junctions; the lines delimited by a junction and one extremity are branches; and the lines delimited by two junctions and a branch are segments for the region shown above. Scaffolds were stained with β-III tubulin (RGCs) in *gray*, S100 β (astrocytes) in *green*, CD31 (ECFCs) in *red*, and dapi to label all nuclei **(A** and **B)** with only the CD31 channel shown in **C**. Scale bar = 2000 µm **(A)**, 500 µm **(B)**, and 100 µm **(C)**.

**Table 1.** Angiogenic Analysis of Vascularized Scaffolds

Extremities	15.1 ± 2.3
Nodes	64.7 ± 8.9
Junction	17.2 ± 2.4
Master junctions	4.9 ± 0.9
Branches	11.9 ± 1.8
Vascularized area	14.6% ± 3.5

Vascularized scaffolds were imaged and analyzed using the Angiogenic Analysis plugin of ImageJ by quarter of each scaffold. Results are reported as the mean ± standard deviation of *n* = 22 analyses.

## Discussion

Organoid models including ROs for investigating human development and physiology have emerged as significant tools for fundamental and translational research. Nevertheless, despite their ability to develop appropriate cellular structures, ROs are unsuitable as a model of glaucoma because of their inability to form a retinal NFL or an ON, nor as a model for diabetic retinopathy due to their inability to form a retinal vasculature. Within the retina, the development of the NFL and the retinal vasculature are linked with cells from the ON head secreting biochemical cues that guide RGCs to the ONH.^54^ These ONHA then further migrate across the RGCs axons leading to the migration of the vascular cells which form the vasculature of the inner neural retina.^17^ While methods of neural guidance in tissue-engineered constructs have been well established through the use of physical guides such as fibers or channels and with soluble or immobilized biochemical cues, vascularization of these constructs and organoids is still a significant issue.^9,11,23,55,56^ The absence of a vasculature limits the thickness, cell density, and complexity of constructs due to nutrient limitations and changes in cell differentiation based upon hypoxic conditions and a lack of secreted factors.^57,58^ While 3D bioprinting has been used to print vasculature into tissue-engineered constructs, the scale of these vessels is often very large and the method is incapable of printing capillary-sized vessels.^59^ This size difference and inability to print the natural patterning of vessels in the inner neural retina could lead to the incorrect organization in the patterning of RGC subtypes and anatomical features such as the fovea whose formation and function require capillary-sized vessels.^60^

In other CNS organoids, the natural mechanism for forming vasculature including capillary-sized vessels has been mimicked through the incorporation of human umbilical vein endothelial cells (HUVEC) cells or fibroblasts that are engineered to be directed to differentiate into endothelial cells.^61,62^ However, these methods use a mixture of cells cultured in a self-organizing fashion and as a result, they are not able to recreate the neural guidance necessary for the formation of the NFL which is integral to the patterning of the vasculature in the inner neural retina. This limitation led to the use of 3D printing to position the astrocyte and endothelial cells within our constructs. Neurons, astrocytes, and endothelial cells have also recently been combined in 3D-printed constructs, however, in this study the different cell types were combined together in a single bioink and not printed individually. While this allows for the printing of macroscopic structures, it does not allow for the correct positioning of the different cells in relation to one another within the printed structure, limiting both the ability to direct the growth of the neurons and the migration of the astrocytes and endothelial cells. This combination of cells into a single bioink limits the overall ability of the printed structure to recapitulate the tissue of interest and ultimately leads to similar results as those without using 3D printing.^63^ In this study, astrocytes and vascular endothelial cells, the two cells that enter the retina through the ON during development, were combined into a single bioink and printed in the center of the radial scaffold while RGCs that originate peripherally to the ON were then positioned on the surrounding fibers of the scaffold. Using 3D printing to mimic the location of these different cells to their corresponding positions within the developing retina allows for the full mechanism of vasculogenesis to occur, including the RGC axon guidance and the astrocyte and endothelial cell migration and ultimately forming a construct that more accurately models the retinal structure.

To facilitate the positioning of the astrocytes on the scaffold, it was necessary to identify a bioink that would support the growth and attachment of both astrocytes and RGCs due to the need for the RGCs to enter the bioprinted structure to facilitate astrocyte guidance. Previously we had utilized matrigel for the 3D printing of RGCs due to the importance of laminin-1 for the survival and neurite outgrowth of RGCs and its presence in the NFL.^64^ Here, we also tested collagen-1 due to its presence in the ON and alginate due to its extensive use in bioprinting.^65,66^ In addition because alginates have been known to have low cell binding, we also tested modified alginate which contained immobilized RGD cell binding domains, and created mixtures of these alginates with the Matrigel and collagen-1.^67^ Finding that matrigel supports the attachment of both RGCs and astrocytes and supports the greatest neurite extension of the RGCs, we used matrigel for the positioning of astrocytes at the center of our scaffolds in all guidance and migration experiments. In our initial studies, we utilized cell lines for types 1 and 2 astrocytes isolated from the cortex of an early postnatal mouse, but in these studies, we did not observe any RGC polarization. However, when we experimented with primary ONHAs isolated from the developing rat retina we observed a significant increase in RGC polarization toward the printed astrocytes. Because it has been previously established that astrocytes are not a uniform cell population with cells in different locations within the central nervous system and from different developmental stages having different functions and expressing different proteins, we sought to determine whether this guidance was limited only to astrocytes from that developmental stage, or from factors only secreted by ONHAs. We therefore isolated astrocytes from the same developmental age but from the surrounding retina not including the ONH and astrocytes from the ONH of adult rats. Interestingly only the ONHAs from the developing retina demonstrated a significant increase in RGC axon polarization while all other astrocytes showed no increase in polarization compared to RGCs cultured on scaffolds where matrigel alone was printed at the scaffold center. The source of these ONHAs from the developing retina could help to explain why RGCs do not extend axons into the organoid center which follow the same developmental stages of the native retina but which lack ONHAs, while the lack of polarization observed with adult ONHAs could also help to explain the limited polarization of RGCs transplanted to adult rodents.

Because electrospun nanofibers have been previously used to guide both glia and endothelial cells, investigation of the next stages of the mechanism of vasculogenesis required the development of an experimental method to distinguish whether that guidance was enhanced by the RGC axons, as is found during retinal development or solely based upon the presence of the electrospun scaffold’s physical guidance cues.^68,69^ To control for potential differences in the electrospinning process and scaffold laminin coatings, RGCs were seeded on half of the scaffold by using a removable PDMS mask, similar to a technique we have used previously,^46^ with the RGCs then allowed to extend their neurites for 48 h prior to the astrocytes and endothelial cells being printed at the scaffold center. Following their printing and culture, P2 ONHA were observed to eventually migrate across the entire scaffold, but these ONHA were observed to migrate to a greater degree on the RGC side of the scaffold as well as migrating further and at a faster rate on this side. This again follows the developmental mechanism and agrees with previous studies using mouse models where RGCs failed to develop in which ONHA migrated to the retinal periphery several days slower than in control animals with RGCs.^17^ ECFCs were also observed to preferentially migrate on RGC axons, though during initial studies prior to media optimization, only minimal migration from the printed matrigel was observed. In addition, those ECFCs which did migrate, migrated as single cells and failed to form continuous networks. However, when samples were cultured in media optimized for the coculture of all three cell types, it was observed that the ECFCs could migrate a greater distance across the scaffold and form vascular networks but importantly these networks were limited only to the halves of scaffolds which contained RGCs. The vasculature formed using this coculture optimized media demonstrated similarities to the developing rodent retina with the endothelial cells within these vascular networks expressing the tight junction marker ZO-1 and having similar numbers of nodes, junctions, and branches as described *in vivo* studies of postnatal mice.^70^ However, it differed from the developing vasculature in that it did not include the larger vessels that extend from the ON head. This is likely due to large vessel formation forming through a separate astrocyte-independent mechanism as is observed in other parts of the CNS.^71^ While 3D bioprinting has been used to create vessels of this size and could be used in the future to form an initial template, large vessel formation may also be observed as this method is translated to ROs which generate tissue-specific ECM which play a part in large vessel templating. There is also a concern that some differences between the vascular patterning in our model compared to the native tissue could be due to xenogenic differences as the RGCs and ONHA originated from rats, while the ECFCs were isolated from humans. While protocols for the differentiation and isolation of human iPSC-derived RGCs and astrocytes have been published and these iPSC-derived astrocytes have been shown to influence the RGC’s maturity, there are currently no protocols for differentiating ONHA specifically.^72–74^ Further, previous work involving human ECFCs has shown the ability of these cells to integrate into the retina of an ischemic mouse model.^48^

Still, it is unclear what signal is causing the axon guidance in the P2 ONHAs which is lacking in the other four astrocyte groups. Similar to other glia, astrocytes secrete a number of factors with those in the retina known to secrete factors such as sparc, Kevin, and thrombospondin as well as regulating BDNF all of which contribute to neuron development, synapse formation, and homeostasis.^75–78^ While astrocytes are known to be involved in neuron guidance, many of the factors associated with this guidance, such as N-cadherin, NCAM, ephrins, slit-1 and 2, and tenascin-C, are either cell surface or ECM bound.^79–83^ In addition, one of the most widely studied soluble guidance molecules in the retina, Netrin-1, is secreted by astrocytes or their progenitors and is known to be necessary for the exiting of RGC axons from the retina through the ON head.^9,15^ Netrin-1 has been shown to enhance axon growth in stem cell-derived RGCs grown in ROs or when transplanted onto retinal explants. However, because this signaling molecule was added to the culture media in these studies it is not clear whether Netrin-1 is capable of directing axon growth to a specific position within the organoid alone. The guidance of RGCs axons by Netrin-1 is complex and its nature as a guidance molecule changes as the retina develops.^6^ As RGCs develop, Netrin-1 is converted from a guidance molecule into a repulsive signal in the presence of Laminin-1, a mechanism we previously observed using immobilized gradients of Netrin-1 on electrospun scaffolds.^11,84^ While this repulsion has been shown to be related to the ratio of two Netrin-1 receptors, DCC and UNC5, in the rodent retina, this conversion occurs before birth. Since the RGCs used in this study were isolated for postnatal rats, and as such should be repulsed by Netrin-1, P2, ONHAs may secrete a factor that alters the expression or function of this mechanism that is not secreted by either the adult populations or the retinal and cortical astrocytes. It is also unclear whether Netrin-1 can function as a long-distance guidance factor which would be necessary as RGC polarization was observed over the entire 1 cm diameter of the electrospun scaffold.^54^ Still, astrocytes do secrete other factors such as BDNF and GDNF which have also been shown to be capable of axon guidance and could be responsible for the increased polarization observed here; however, it should be noted that the media used in these studies contains 50 ng/mL BDNF which could mask its secreted effect. Ultimately, differential proteomic analysis of the secretomes from these different astrocyte populations may help identify the factors responsible for this increased guidance.

## Conclusions

We have demonstrated in this study that astrocytes isolated for the ON head of the developing retina express factors that are capable of increasing the polarization of RGCs toward our construct’s center, where these astrocytes have been printed. In contrast, astrocytes isolated from the retina at the same developmental time point, from the adult ON head, or astrocyte cell lines from the cortex at the same developmental time point do not increase RGC polarization. We have shown that ONHAs printed in our system migrate to a greater extent as well as further on RGC axons consistent with observations of *in vivo* astrocyte migration. Finally, we have demonstrated that vascular endothelial cells printed with ONHA will preferentially migrate along RGC axons and that these cells only form vascular networks on the parts of scaffolds on which RGCs have been seeded. Taken together, we have demonstrated that by combining 3D bioprinting with electrospun scaffolds we are able to recreate the mechanism of vasculogenesis observed in the developing retina. In this study, we have demonstrated a method for organizing RGC growth and polarization that is currently missing from RO and other models of glaucoma and which can be expanded to other central nervous system models or tissue-engineered replacements in the future.
