# Supplementary material for: Three-Dimensional Bioprinting of Astrocytes and Endothelial Cells to Direct Retinal Axon Growth and Vascularization
Source: Tissue Eng Part A. 2025 Nov 14;31(21-22):1253–68. doi: 10.1089/ten.tea.2024.0326 (PMC12704128; doi:10.1089/ten.tea.2024.0326)
Supplement: Supplementary file 1 [file ten.tea.2024.0326_supplementary_data.pdf]

Supplemental Figure 1: Labelling of RGC Axons

RGCs were cultured on radial scaffolds in RGC optimized media for 3 days and then stained for β3 tubulin (green), which labels both axons and dendrites, and SMI 312 (red) which labels axons and dapi to label all nuclei (blue). All neurites greater than two cell bodies in length, the criteria used in axon growth assays, stained positive for both markers. Scale bar 100μm.

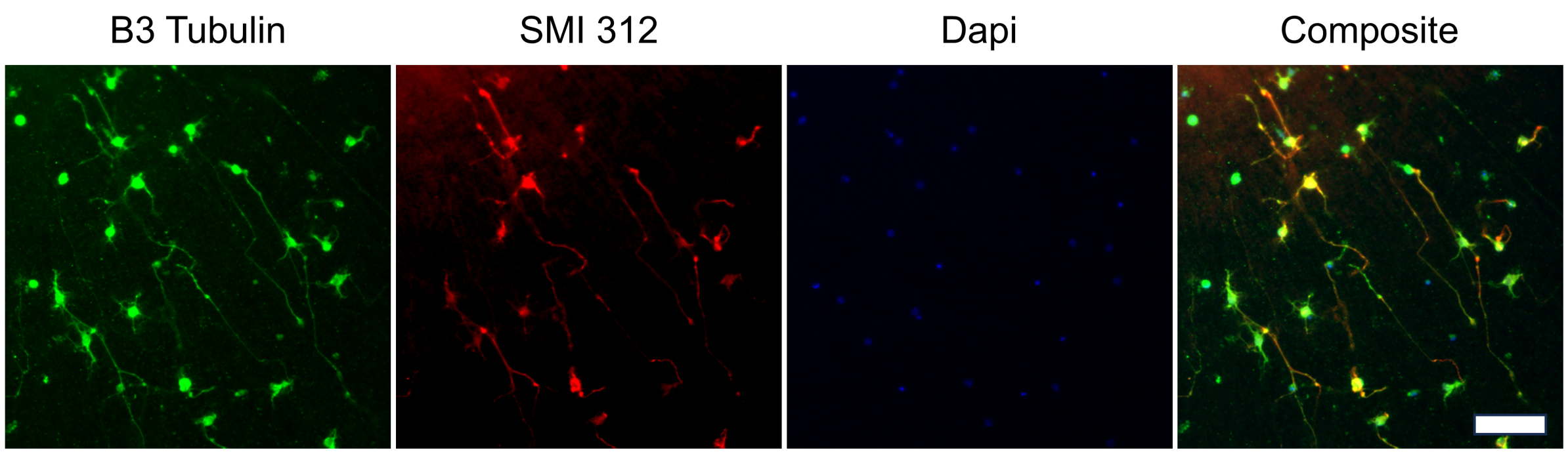

Supplemental Table 1. Media Components

Media components used in the different culture media including previously optimized RGC media (A), RGC media supplemented for astrocyte co-cultures (B) and EGM-2 growth factors used in optimized media. EGM-2 growth factors in EGM-2 base media (Lonza) with an additional 2% FBS were used for the initial expansion of ECFCs. Concentrations in C are based upon values published by Lonza.

A.

RGC Optimized Media<br>Made in Neurobasal Media	
**Component**	**Final Concentration**
B27- Plus	1x
BDNF	50 ng/ml
BSA	100ug/ml
CNTF	10 ng/ml
Forskolin	5 µM
Glutamax	2 mM
Insulin	5 µg/ml
N-acetyl cysteine	5 µg/ml
Pen/Strep	1x
Progesterone	0.2 µM
Putrescine	16 µg/ml
Sodium Pyruvate	1 mM
Sodium Selenite	40 ng/ml
Transferrin	100 µg/ml
Triiodo-thyronine	0.06 µM

B.

Astrocyte Supplemented Media<br>Made in RGC Optimized Media	
**Additional Components**	**Final Concentration**
HBEGF	10 ng/ml
N2 Supplement	1x

C.

EGM-2 Growth Factors	
**Components**	**Final Concentration**
Hydrocortisone	20 ng/ml
hFGF-B	4 ng/ml
VEGF	2 ng/ml
R3-IGF-1	5 ng/ml
Ascorbic Acid	75 µg/ml
hEGF	20 ng/ml
Gentamicin	30 µg/ml
Amphotericin	15 ng/ml
Heparin	1 ng/ml

Supplemental Table 2. Antibodies Used for Purification

Antibodies and catalog numbers for the purification of RGCs (A), subpopulations of ONH Cells (B) and Endothelial Colony Forming Cells (C).

A.

Antibodies for RGC Immunopanning	
**Antibody**	**Catalog**
Anti-Macrophage	Accurate Chemical and Scientific Corp AIA51240
Anti-Thy1.1	ATCC T11D7e2

B.

Antibodies for Optic Nerve Head Cell Immunopanning	
**Antibody**	**Catalog**
BSL-1	Vector Laboratories L11005
Anti-Rat IgG	Jackson Immuno Research Labs 112005167
Anti-CD45 (Ox1)	BD Biosciences 554875
Anti-O4	R&D Systems FAB1326A
Anti-ITGB5	R&D Systems AF8035
Anti-C5	Developmental Studies Hybridoma Bank C5/A4
Anti-CD13	Proteintech Group Inc 145531AP150UL

C.

Antibodies for Endothelial Colony Forming Cell Purification	
**Antibody**	**Catalog**
Anti-CD31	BD Biosciences 555446
Anti-CD45	Thermo Scientific MA513197
Anti-CD90	BD Biosciences 550402
